# Down syndrome birth rate post Dobbs decision: has it changed?

**DOI:** 10.1038/s41372-026-02560-w

**Published:** 2026-02-02

**Authors:** Stephanie L. Santoro, Chance Alvarado, Stephanie Y. Tseng, Sara Conroy, Isaac Kistler, Stephen A. Hart, Samantha Fichtner, Clifford L. Cua

**Affiliations:** 1 Massachusetts General Hospital, Boston, MA USA; 2 Nationwide Children’s Hospital Heart Center, Nationwide Children’s Hospital, Columbus, OH USA; 3 Center for Biostatistics, The Ohio State University, Wexner Medical Center, Columbus, OH USA; 4 Biostatistics Resource at Nationwide Children’s Hospital, Abigail Wexner Research Institute, Nationwide Children’s Hospital, Columbus, OH USA; 5 Center for Perinatal Research, Nationwide Children’s Hospital, Columbus, OH USA

**Keywords:** Epidemiology, Health policy

## Abstract

**Objective:**

In 2022, the U.S. Supreme Court decision on Dobbs v. Jackson Women’s Health Organization changed abortion access. We evaluated birth rates of Down syndrome (DS) before and after the Dobbs decision hypothesizing similarity to cyanotic congenital heart disease.

**Study design:**

Retrospective cohort study of live births utilizing 2016 – 2025 CDC birth certificate registry data stratified by DS and by state abortion access categories (restrictive and protective) based on the Guttmacher Institute. Incidence of DS and the difference in incidence between state cohorts were modeled and compared between pre- and post-Dobbs periods using segmented linear regression.

**Results:**

Incidence of DS live births remained stable in restrictive and protective states pre- and post-Dobbs. Segmented regression models revealed no significant changes in monthly incidence in the post-Dobbs era nationally compared to pre-Dobbs.

**Conclusions:**

Mean monthly incidence of DS in live-born infants using CDC birth certificate data did not increase after the Dobbs decision.

## Introduction

Down syndrome (DS) is the most common and viable human trisomy, with an incidence of approximately 1 per 700 live births in the United States [1]. Patients with DS have an increased risk of anatomical as well as physiologic abnormalities compared to the general population, which places them at increased risk of morbidity and mortality [2–15]. The rate of pregnancy termination following a diagnosis of DS in longitudinal studies is variable and likely dependent on multiple factors that cannot be fully accounted for in these studies [16–21].

In June 2022, *Dobbs v. Jackson Women’s Health Organization* ruling by the U.S. Supreme Court resulted in abortion policies being decided at the state rather than the federal level. This ruling led to variations between states in access to pregnancy termination. Increases in birth rates for cyanotic congenital heart disease (CCHD), specifically, and congenital anomalies, in general, have been documented in states with restrictive abortion policies compared to states with more protective policies post-*Dobbs* [22–24]. In addition, neonatal and infant death rates were also noted to increase in restrictive states vs less stringent states [22–24]. The impact of the *Dobbs* decision on the birth rate of patients with DS has not been definitively evaluated.

The goal of this study was to document birth rates of infants with DS before and after the *Dobbs* decision and to assess for differences in DS birth rates by presence of restrictive and protective abortion policies.

## Methods

### Study Population

U.S. states were assigned one of seven levels of abortion access, as depicted by an interactive graphic from Guttmacher Institute as previously described [24, 25]. Levels of protection or restriction were assigned based on state-level policies, including bans, waiting periods, constitutional protections, insurance coverage, and clinic staff protections as previously described [24, 26]. We further categorized births in states with the highest two levels of abortion restriction, very restrictive and most restrictive, into a single restrictive cohort. Births in states with the highest two levels of abortion protection, very protective and most protective, were assigned to a comparison protective cohort. Upon access of the Guttmacher interactive graphic on July 17, 2024, this categorization yielded 20 and 9 states in the restrictive and protective cohorts, respectively. This categorization has not shifted significantly since the *Dobbs* decision (Table 1).

**Table 1:** States in Restrictive and Protective Cohorts.

Cohort*	States				
**Restrictive**	Alabama	Arizona	Arkansas	Florida	Georgia
	Idaho	Indiana	Kentucky	Louisiana	Mississippi
	Missouri	Nebraska	North Dakota	Oklahoma	South Carolina
	South Dakota	Tennessee	Texas	Utah	West Virginia
**Protective**	California	Maryland	Michigan	Minnesota	New Jersey
	New Mexico	New York	Oregon	Vermont	

^*^Cohort designation based on the Guttmacher Institute interactive map (https://states.guttmacher.org/policies/).

Information related to live births was obtained from the publicly available *Natality Information – Live Births* databases hosted by Centers for Disease Control and Prevention (CDC) Wonder [10, 11]. These databases are sourced from birth certificate data and consist of live births occurring within the U.S. Finalized data for 2016–2022 were retrieved alongside 2023–May 2025 provisional data for all recorded births and births from restrictive and protective states. Counts of live births pulled from CDC Wonder were stratified by month and year. DS diagnosis was not available in the data prior to 2016. In the CDC Wonder dataset, individuals can be classified with a diagnosis of DS as either confirmed, pending, no, or unknown. Datasets were obtained as public-use data files and accessed on June 18, 2025; sample size was chosen to include all eligible individuals in the CDC Wonder dataset.

### Ethics approval and consent to participate

All methods were performed in accordance with the relevant guidelines and regulations. The Institutional Review Board determined this research was not considered human research and approval was not required. As such, we were not required and did not obtain informed consent from the participants in the CDC Wonder dataset, but rely on publicly-available datasets of de-identified information.

### Exposures and Outcome

The primary exposure was the June 24, 2022, *Dobbs v. Jackson Women’s Health Organization* decision by the U.S. Supreme Court. Natality information could only be queried at the month fidelity, and thus, July 2022 was considered the first data point exposed to the *Dobbs* decision.

The event of interest was confirmed or pending DS diagnosis in live births. Due to varying numbers of births by state cohorts and the widely observed seasonality of births, incidence rates relative to monthly births at the national and state cohort level were calculated [27]. Those with unknown or not stated DS diagnosis were excluded from both the numerator and denominator during the calculation of incidence rates.

### Statistical Methodology

As in our previous work [24], observed national and state cohort-level trends in monthly incidence of live births with DS were visualized alongside smoothed trends from a centered four-month moving average. The difference in monthly incidence of DS between restrictive and protective cohorts was calculated such that a greater value represents a higher incidence in restrictive states. Incidence rates were additionally scaled to rates per 100,000 live births for ease of interpretation.

Segmented linear regression models were implemented to quantify potential changes in the incidence of DS pre- and post-*Dobbs*, nationally, in protective and restrictive state cohorts, and among differences in monthly incidence between cohorts. Models were specified such that estimates represent the monthly change in incidence of confirmed/pending DS during the pre-*Dobbs* period as well as the post-*Dobbs* change in monthly incidence (slope/change in slope) [28]. No immediate level change at the *Dobbs* decision was estimated, given the assumption that changes in DS incidence would gradually increase as more pregnant mothers considering abortion were impacted by state-level policies enacted in response to the *Dobbs* decision.

Validity of the modeling framework was assessed via examination of residuals over time and Q-Q (quantile-quantile) plots. Model estimates were scaled to rates per 100,000 live births and presented alongside 95% confidence intervals and *p*-values. The estimated pre-*Dobbs* linear trend was superimposed over realized monthly incidence for the entire analysis period, representing the hypothetical continued trend in the absence of the *Dobbs* decision (counterfactual). The estimated post-*Dobbs* trend was visualized alongside 95% confidence intervals.

Analysis and reporting of results were in alignment with the Strengthening the Reporting of Observational Studies in Epidemiology (STROBE) reporting guidelines [29]. All statistical analyses were performed using R version 4.2.2 [30].

## Results

### Observed Trends in Incidence

Nationally, the monthly incidence rate (mean, standard deviation (SD)) of births with DS over the study period was 53 (4.3) per 100,000 live births; pre-*Dobbs*, the rate was 53.8 (4.2) per 100,000 live births and post-*Dobbs*, the rate was 51.2 (4.3) per 100,000 live births.

**Fig. 1:**
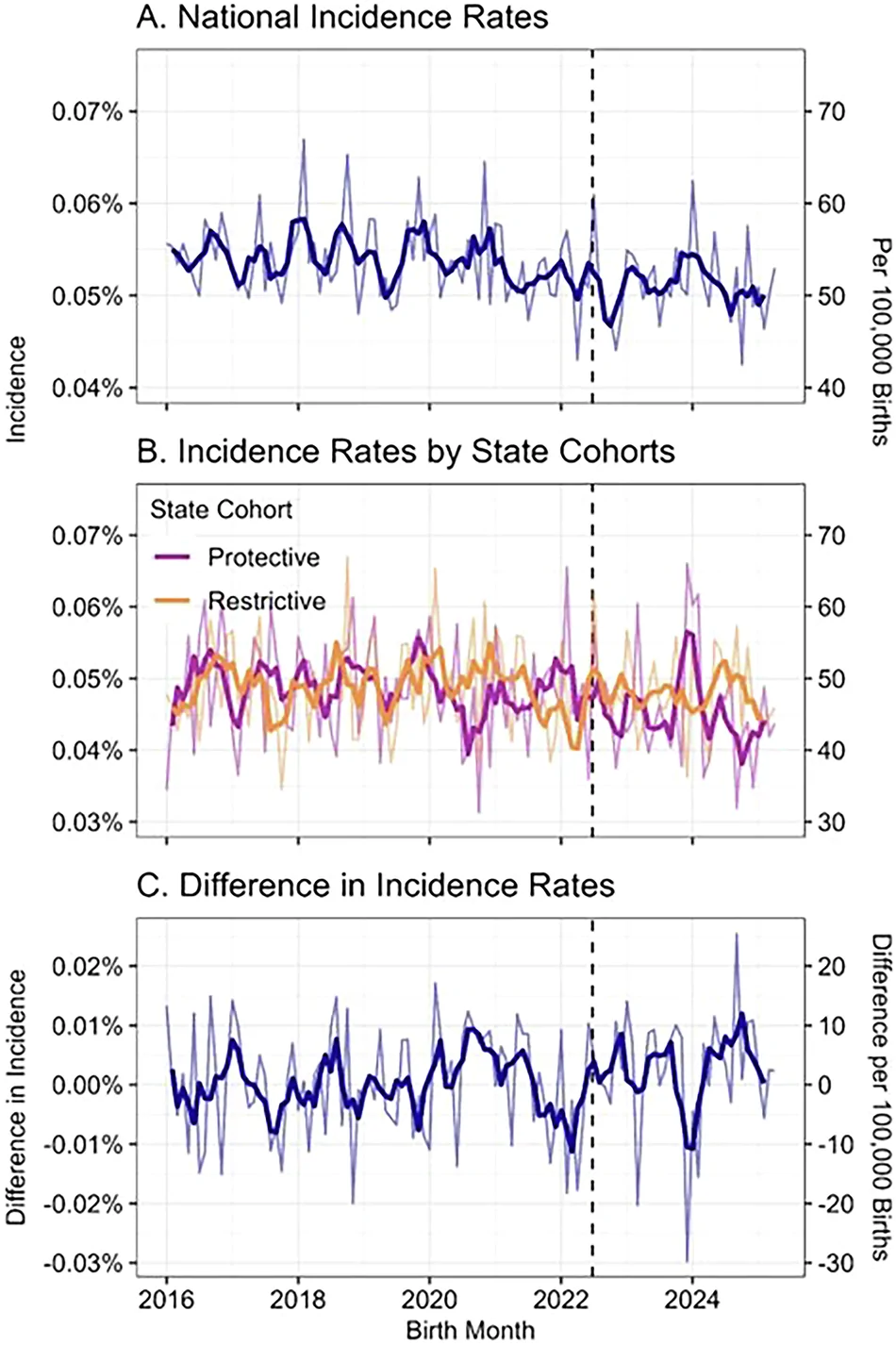
Incidence of confirmed/pending Down syndrome diagnosis nationally and by state cohort. Incidence of confirmed/pending Down syndrome diagnosis (**A**) nationally, (**B**) by state cohorts, and (**C**) difference between state cohorts over time. Dashed vertical line represents the *Dobbs* decision. Thinner lines represent observed incidence in respective cohorts while thicker lines represent centered four-month moving average.

Figure 1 illustrates the observed and four-month smoothed trends in confirmed or pending DS incidence at birth (A) nationally, (B) by state cohort, and (C) the difference in incidence between state cohorts. Despite fair month-to-month variability, longer-term trends appeared stable, and visually there was no indication of a change in incidence associated with the post-*Dobbs* period. Protective and restrictive states showed similar monthly incidence rates across the observation period. The mean (SD) monthly incidence pre-*Dobbs* was 48.5 (7.1) and 48.6 (6.7) per 100,000 live births in protective and restrictive states, respectively. Post-*Dobbs*, DS incidence rates per 10,000 births were 45.6 (7.8) and 48.1 (5.8) in protective and restrictive states, respectively.

### Segmented regression analysis and hypothetical Dobbs-free incidence rates

**Fig. 2:**
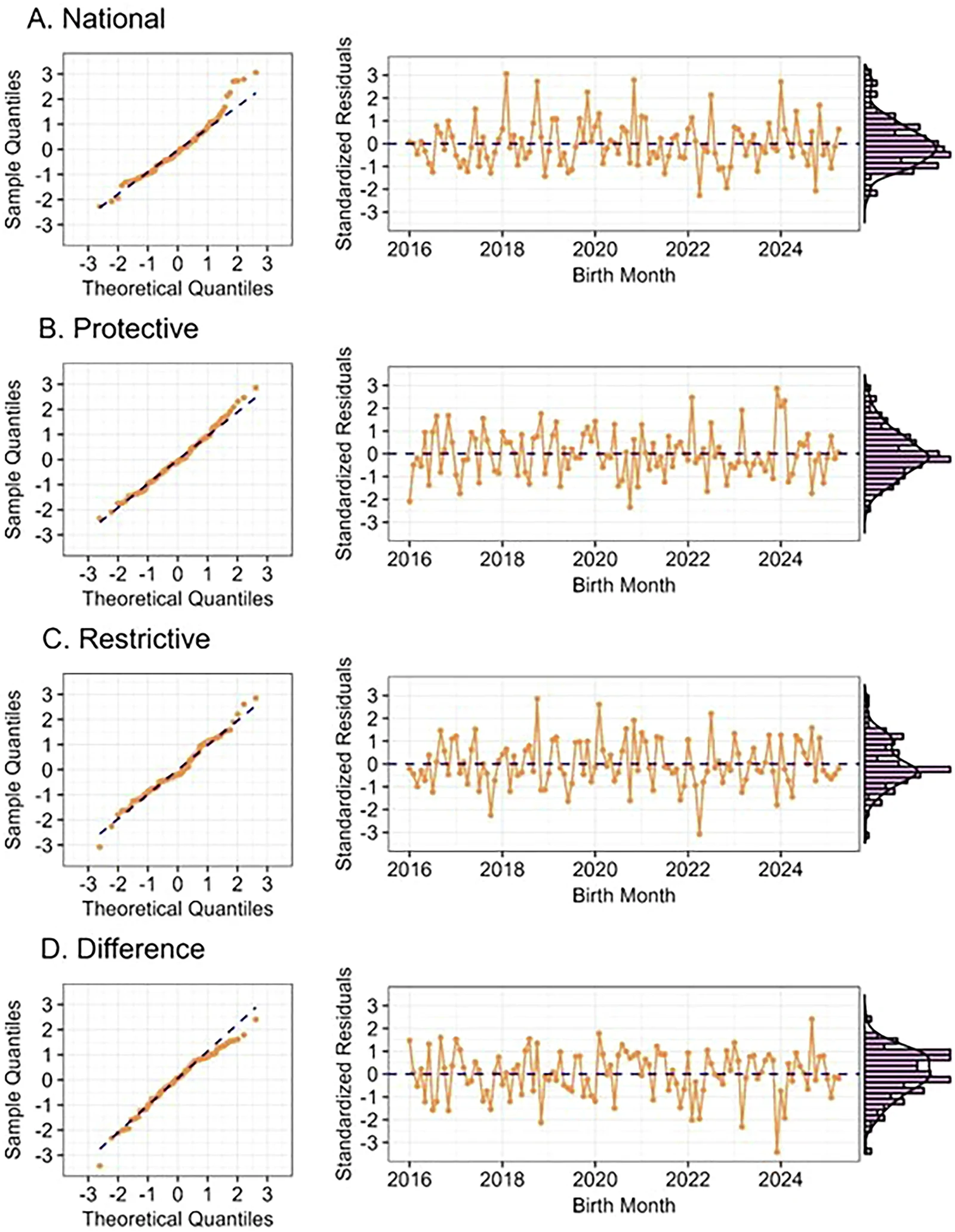
Diagnostic Plots for Segmented Regression Models. Q-Q (quantile-quantile) plots (left) and standardized residuals over time (right) for linear regression models (**A**) nationally, among (**B**) protective and (**C**) restrictive states, and (**D**) the difference in incidence between state cohorts. Densities of standardized residuals are additionally presented along the right axis of the rightmost panels.

Segmented linear regression models were implemented to further explore changes in the incidence of confirmed or pending DS at birth nationally, in protective states, in restrictive states, and in monthly differences in incidence between state cohorts. Via examination of residuals, the models appeared to explain the data well (Fig. 2).

Table 2 notes model estimated coefficients describing the average pre-*Dobbs* monthly increase/decrease in incidence and the change in slope associated with the post-*Dobbs* period. Nationally, a slight downward trend in incidence of DS was noted with a monthly change in incidence of −0.04 (95% CI: −0.076, −0.005; *p* = 0.03) per 100,000 live births. In the post-*Dobbs* period, this rate of change remained constant with a change in slope of −0.01 (95% CI: −0.136, 0.115; *p* = 0.87) estimated. At the protective and restrictive state cohort level, there was no observed decrease in monthly incidence pre-Dobbs. There was weak evidence that in the post-*Dobbs* period, protective states experienced a decreasing trend in monthly incidence relative to their pre-*Dobbs* trend (change in slope −0.118; 95% CI: −0.337, 0.102; *p* = 0.29). The monthly change in the difference in incidence between state cohorts remained constant during the pre-*Dobbs* era. While an increasing difference in incidence was estimated in the post-*Dobbs* era, thought to be driven by the decrease in estimated incidence in protective states, the 95% confidence interval and associated *p*-value provided little evidence supporting this effect (95% CI: −0.193, 0.378; *p* = 0.52).

**Table 2:** Pre-Dobbs Monthly Increase/Decrease in Incidence and Post-Dobbs Difference in Down syndrome births.

Cohort	Term	Change in Incidence per 100,000 Live Births (95% CI)	*p*-value
**National**	Pre-Dobbs Monthly Increase/Decrease	−0.04 (−0.076, −0.004)	0.03
	Change in Slope from Pre-Dobbs Period	−0.01 (−0.136, 0.115)	0.87
**Protective**	Pre-Dobbs Monthly Increase/Decrease	−0.019 (−0.083, 0.044)	0.55
	Change in Slope from Pre-Dobbs Period	−0.118 (−0.337, 0.102)	0.29
**Restrictive**	Pre-Dobbs Monthly Increase/Decrease	−0.009 (−0.065, 0.047)	0.75
	Change in Slope from Pre-Dobbs Period	−0.025 (−0.219, 0.169)	0.80
**Difference**	Pre-Dobbs Monthly Increase/Decrease	0.01 (−0.072, 0.093)	0.80
	Change in Slope from Pre-Dobbs Period	0.093 (-0.193, 0.378)	0.52

**Fig. 3:**
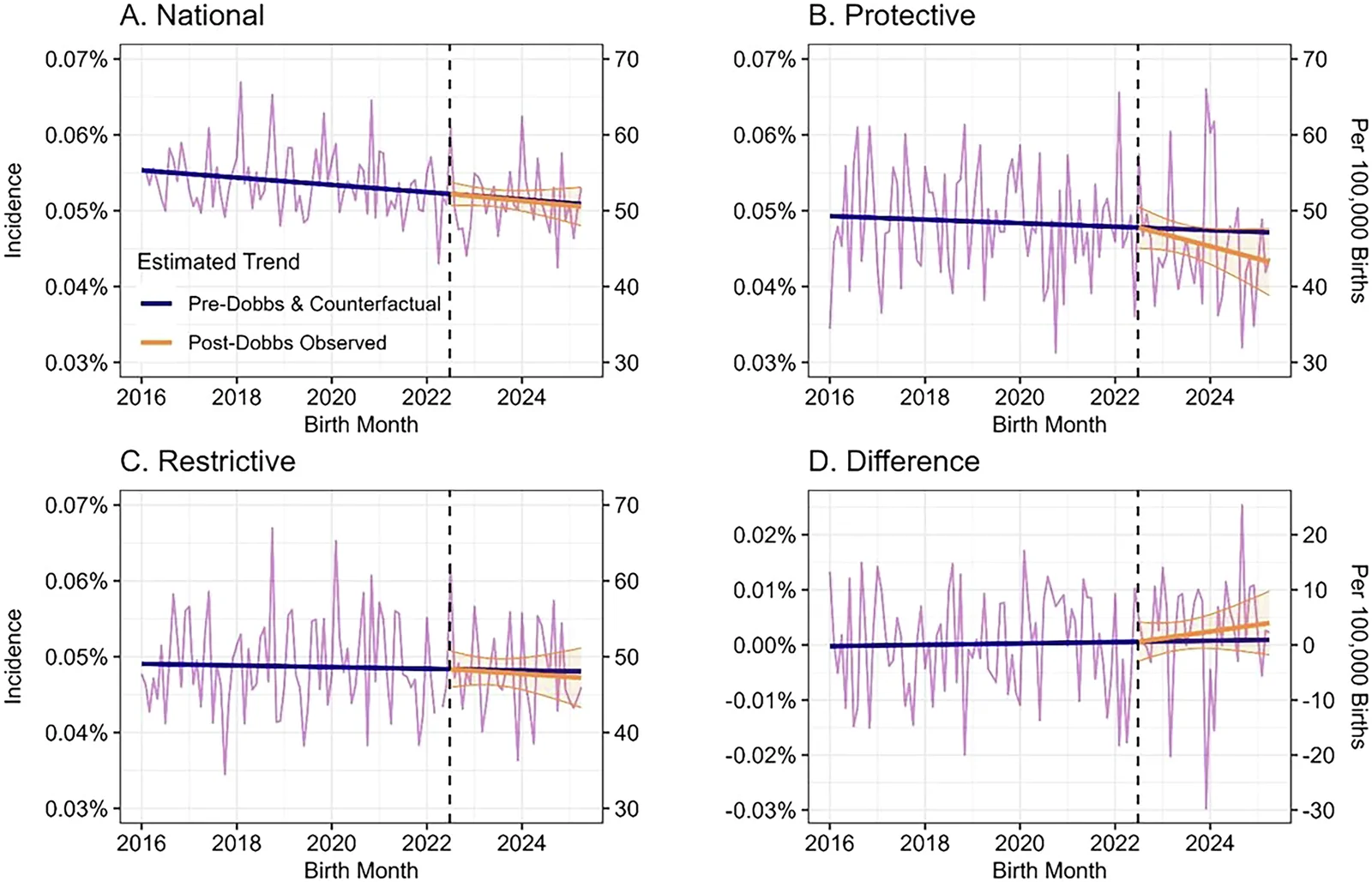
Observed and hypothetical Dobbs-free trends nationally and by state cohort. Estimated linear trend (thicker, purple line) in incidence of confirmed/pending Down syndrome diagnosis in the pre-Dobbs period and its hypothetical continuation in the absence of the Dobbs decision is shown compared to the estimated post-Dobbs trend (orange line and shaded region representing 95% confidence interval) (**A**) nationally, among (**B**) protective and (**C**) restrictive states, and (**D**) the difference in incidence between state cohorts. Dashed vertical line represents the *Dobbs* decision. Observed monthly incidence is shown in each panel as a thinner, lighter line. In the panel representing the difference between state cohorts (**D**), positive values indicate greater incidence in restrictive states compared to protective states.

Figure 3 superimposes the estimated pre-*Dobbs* trends from the segmented linear regression models across the entirety of the observed time series. In the post-*Dobbs* period, this trend represents hypothetical incidence rates had the *Dobbs* decision not been enacted. The change in trends based on realized post-*Dobbs* rates was also presented alongside 95% confidence intervals. As expected, visualized trends are consistent with the interpretation of model coefficients presented in Table 2. Despite an estimated decrease in incidence of DS in protective states during the post-Dobbs period and a subsequent increase in monthly differences between state cohorts, the 95% confidence intervals are wide and encompass the hypothetical *Dobbs*-free counterfactuals.

## Discussion

To-date, the impact of the *Dobbs* decision on the birth rate of individuals with DS has not been evaluated. Through review of live birth datasets available from the CDC [24], we conducted modeling to determine if the incidence of DS had changed nationally, in protective and restrictive state cohorts, or between cohorts after the *Dobbs* decision in June 2022. We found that national birth rates of infants with DS before and after the *Dobbs* decision did not significantly differ. This unchanged birth rate was also confirmed in state subgroups classified by restrictive and protective abortion policies.

We conducted this study to evaluate for changes in birth rates of infants with DS after the recent *Dobbs* decision. As outlined, the *Dobbs* decision impacted prenatal care options and we hypothesized that decreased access to abortion care could lead to fewer terminations and, thus, an increased rate of liveborn infants with DS. In past work, similar hypotheses were examined for CCHD, with findings supporting an increase in CCHD incidence overall and specifically in restrictive states [24]. Total and state-based (i.e., in states with protective and restrictive abortion policies) birth rate changes that were seen in CCHD were not observed in this study of individuals with DS. We theorized that CCHD and DS would show similar post-*Dobbs* changes in birth rates as both are: often diagnosed prenatally with screening tools [31] that are relatively non-invasive (i.e., through routine screening, such as cell-free fetal DNA (cffDNA) or ultrasonography) and which should be offered to all patients [32]. Thus, we hypothesized that evaluation for CCHD and DS would have similar access, availability, and uptake. Further, prenatal testing for CCHD and DS, which are medically complex conditions, may lead to discussion of available reproductive options. For example, with abnormal prenatal genetic testing results, it would be common for a practicing clinician to present a range of options depending on many factors (such as a family’s values, the health of the mother, the potential impacts on mother’s health, the severity of the current findings, and the prognosis of the condition, among others) and to offer referral to a genetic counselor or to a multidisciplinary fetal care center depending on the complexity of the findings.

Despite these theoretical similarities, we did not find the expected changes in birth rates of infants with DS that we found for infants with CCHD post-*Dobbs*. This could be due to several factors. First, pregnant women and families may perceive the prenatal diagnosis of DS “differently” than a prenatal diagnosis of CCHD. Qualitative research has found that parents of individuals with DS describe feelings of “rescuing hope” and a social process of sense-making with recalibration of expectations for the future at time of diagnosis [33]. Qualitative research in CCHD found that parents perceived challenges related to needing repeated studies for accurate diagnosis or management, insufficient information provided about the CCHD diagnosis and lack of cardiologist present at time of initial CCHD diagnosis [34]. If prenatal diagnosis of CCHD and DS are perceived in different ways, this may impact how the information is acted on in regards to pregnancy termination. Future studies could evaluate the nuances of these prenatal discussions. In fact, literature review of fourteen studies in various countries found that termination rates following the detection of DS by non-invasive pregnancy testing (NIPT) were unchanged or decreased compared to historical termination rates [18]. Those results are similar with this study’s findings of no significant change in DS birth rates post-*Dobbs*. Future study could also explore live birth rates, rates of elective termination, and rates of stillbirth though this data is only available through some state datasets on a smaller scale.

Additionally, it is possible that there are differences between CCHD and DS in the steps leading up to that prenatal discussion. For example, we hypothesized that prenatal testing would have similar uptake, but it might be that another factor (e.g., insurance coverage of a routine ultrasound vs cffDNA, patient comfort with completing ultrasound vs cffDNA even if offered, or anticipation of completing tests as ultrasounds may be an exciting time to “see” the baby for the first time whereas cffDNA is an additional blood test) leading to differences in prenatal decision making that we have not considered.

Those with children with DS and CCHD might differ on a demographic trait, which may confound our analysis. For example, the age of the mother impacts the likelihood of child having DS [35]; it is possible that mothers of infants of individuals with DS are older than mothers of children with CCHD. Additionally, geographic differences have been seen in rates of live births and elective terminations in the past, pre*-Dobbs* [36]. Although our data was collected from a national database through the CDC, our data was de-identified, in aggregate, and does not allow exact correspondence between a birth and the infant’s or mother’s, demographic traits such as maternal age, nor does it allow for evaluation of differences within states or by other factors.

It would be interesting to evaluate the subset of infants who have both a DS and CCHD diagnosis (DS + CCHD), and determine if the birth rates of DS + CCHD resemble those with CCHD or those with DS, or if they are different than both groups. This was not within the scope of the primary aim of this study. Further, it would be interesting to track how the birth rates of individuals with DS + CCHD change longitudinally as past research has demonstrated that rates of certain types of heart defects in DS have changed over time [37]. Regardless, future studies would be helpful to understand the differences between the findings in this study of infants with DS and the prior study of prenatally-diagnosed CCHD, for example, the type of heart defect and its presentation could be important to explore but was not feasible through the data available in this study.

Although our results were obtained from a large national database through the CDC, our study is limited by the type of data collected and recorded there. Given our findings of lower birth rate prevalence and no change in incidence post-*Dobbs* as we hypothesized, future studies will be particularly valuable to replicate these findings and to understand why the incidence of DS in live births has not been impacted by *Dobbs*. Our source was birth certificate data, and it is possible that the diagnosis of DS may not be identified immediately after birth and diagnosed later, thus we may be missing individuals with DS in our cohort; we could have been missing babies who had DS but were not actually noted to have it on the birth certificate. In our study, we included those diagnosed with DS and those with a pending diagnosis of DS in our calculations to try to mitigate this. We did not include those with DS listed as “unknown” which could impact our raw values (i.e., birth rate) but would not be expected to impact our longitudinal comparisons before and after *Dobbs*, as we used the same criteria over time.

## Conclusions

The monthly incidence of DS in live born infants using CDC birth certificate data did not increase after the *Dobbs* decision and was likely not affected by changes in abortion access. Further studies will be important to confirm this trend and to evaluate how a prenatal diagnosis of DS differs from a prenatal diagnosis of other congenital anomalies in influencing management of the pregnancy.

## Data Availability

Statement: The datasets generated during and/or analysed during the current study are available in the CDC Wonder repository, https://wonder.cdc.gov/.
